# Post-infarction ventricular septal rupture with a contained right ventricular pseudoaneurysm formation

**DOI:** 10.1259/bjrcr.20210129

**Published:** 2021-10-17

**Authors:** Giovanni Melina, Tiziano Polidori, Damiano Caruso, Carlotta Rucci, Giuseppe Tremamunno, Roberto Bianchini, Camillo Autore, Andrea Laghi

**Affiliations:** 1 Cardiac Surgery Unit, Department of Clinical and Molecular Medicine, Sapienza University of Rome - Sant'Andrea University Hospital, Rome, Italy; 2 Radiology Unit, Department of Surgical and Medical Sciences and Translational Medicine, Sapienza University of Rome - Sant'Andrea University Hospital, Rome, Italy; 3 Cardiology Unit, Department of Clinical and Molecular Medicine, Sapienza University of Rome - Sant'Andrea University Hospital, Rome, Italy

## Abstract

Mechanical complication of acute myocardial infarction, such as left ventricular free-wall or septal rupture, pseudo-aneurysm or true aneurysm, are uncommon but potentially fatal conditions, that require an early diagnosis and management. We describe a case of post-infarction ventricular septal rupture with pseudoaneurysm formation included in the right ventricle.

## Case presentation

A 75-year-old male with past medical history of hypertension and hyperlipemia, presented with eight days of dyspnea on exertion and chest pain. Triage electrocardiogram (ECG) and laboratory findings were suggestive of a posterior wall MI.

## Investigations

**Figure 1.**
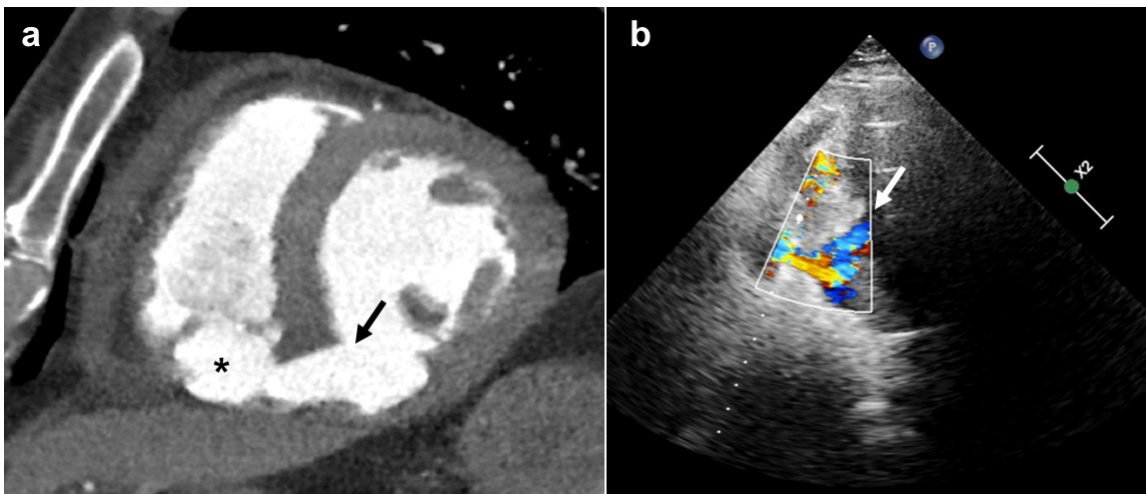
Imaging demonstration of VSD. (**a**) CTTA biventricular short axis view showing an inferior and inferoseptal LV wall defect of mid-ventricular cavity (black arrow) and an oval cavity included in the RV (black asterisk). (**b**) TTE shows the LV to RV shunt through the LV wall interruption (white arrow). CCTA, cardiac CT angiography; LV, left ventricle; RV, right ventricle; TTV, transthoracic echocardiography; VSD, ventricular septal defect.

A trans-thoracic echocardiogram (TTE) showed severe left ventricular (LV) dysfunction and the presence of a pulsatile chamber (maximum dimension 40 × 20 mm) at the apex of the right ventricular cavity, adherent to the free wall. This chamber was connected to the LV cavity through a postero basal septal defect and was supplied during the systolic phase (Figure 1). The patient was taken for cardiac catheterization and found to have a proximal occlusion of the right coronary artery that was successfully treated with Percutaneous Coronary Intervention. Due to severe heart failure, an Intra Aortic Balloon Pump (IABP) was implanted under fluoroscopy guidance through a percutaneous left femoral access. He was then transferred to the Coronary Care Unit with adequate medications including subcutaneous low-molecular weight heparin and dual anti platelet agents.

When clinical stability was reached, an ECG-gated cardiac CT angiography (CCTA) was performed on a 256-slice CT scanner (Brilliance iCT, 256-slice, Philips Healthcare, Best, The Netherlands) to confirm the echocardiographic findings and have a more accurate imaging.

The remaining scanning parameters were set as follows: reference tube voltage, 100 kVp; reference tube current, 300–500 mAs; gantry rotation time 0.27 s; pitch, 0.16; and detector configuration, 128 × 2 x 0.625 mm.

CCTA was performed using a bi-phasic contrast injection protocol with a bolus of 70 ml of contrast media (Iomeprol 400 mg Iodine/mL; Iomeron 400; Bracco Imaging, Milan, Italy) injected at a rate of 4.5 ml s^−1^ followed by 40 ml of saline chaser bolus injected at the same flow rate. We applied the following bolus-tracking protocol: the trigger threshold was set at the level of the tracheal bifurcation; when the pulmonary arteries reached 100 Hounsfield Unit (HU), patients were instructed to breathe in and hold, and scanning was initiated once the contrast bolus reached the ascending aorta (trigger level of 150 HU).

CT scans showed a wide ventricular septal defect (VSD) in the mid-cavity inferior and inferoseptal LV wall, which resulted in a pathological communication with an oval cavity included in the right ventricle (RV) and contained by a thin external layer of RV endocardium.

**Figure 2.**
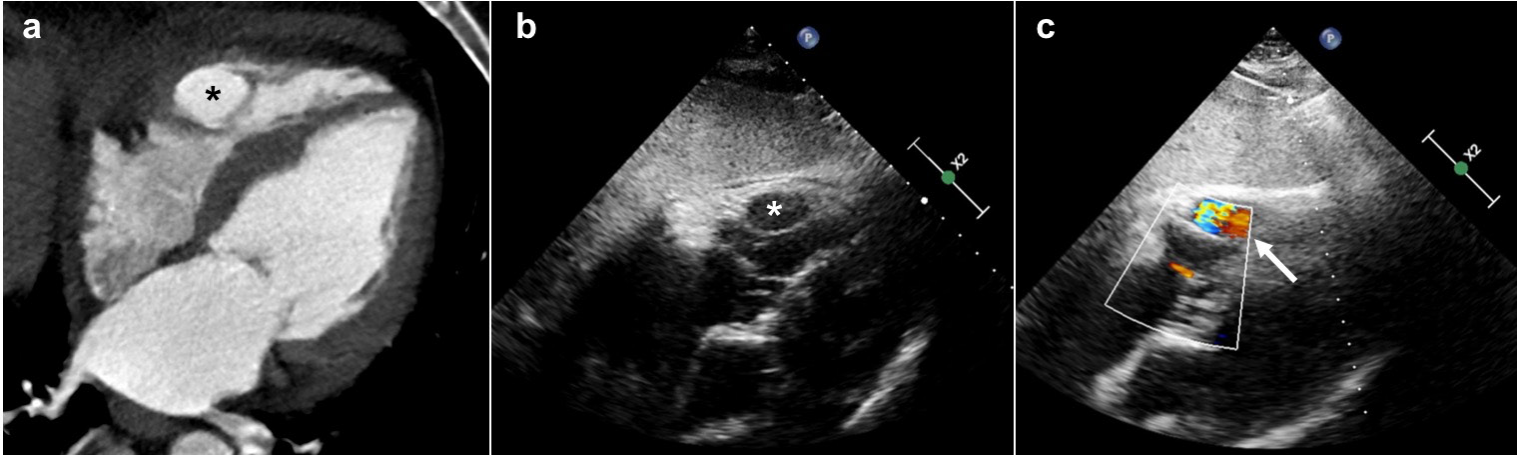
Right ventricle pulsatile chamber (**a–b**) CCTA and TTE four-chamber view showing the RV intracavitary pulsatile chamber (asterisks). (**c**) Color-doppler four-chamber view showing the blood flow of the oval cavity contained inside the RV (white arrow). CCTA, cardiac CT angiography; RV, right ventricle; TTE, transthoracic echocardiography.

The retrospective ECG-gating allowed the acquisition of all cardiac phases and made possible the kinetic analysis of the VSD and the newly formed RV chamber. The VSD became larger during systole, and smaller during diastole. The pulsatility with systolic expansion of the RV chamber was confirmed; the external layer of RV endocardium which included the chamber seemed to be interrupted, defining a pathological inter ventricular communication (Figures 1–2).

In accordance with these findings, we defined the pulsatile chamber as a post-infarction pseudoaneurysm of the interventricular septum (IVS) expanded and ruptured inside the right ventricular cavity.

## Treatment

**Figure 3.**
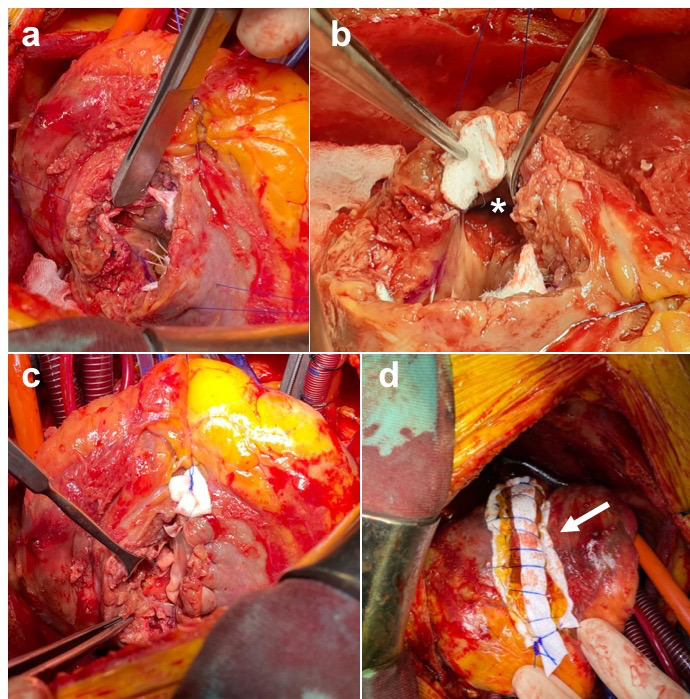
Surgical closure of the septal defect. (**a–b**) The wide septal defect was identified (with asterisk) and the pericardial patch was anchored to the LV. (**c–d**) Images show the final LV closure with VSD exclusion (white arrow). LV, left ventricle; VSD, ventricular septal defect (VSD)

After 2 weeks since presentation, the patient underwent a successful surgical VSD repair using the patch exclusion technique. Figure 3a and b showed the wide septal defect and the pericardial patch anchored to the left ventricle and the final LV closure with VSD exclusion (Figure 3c and d). The patient made an uneventful recovery, IABP removed on Day 3 and he was fit to go home on Day 7 post-operatively. Pre-discharge echocardiography showed an excellent result with only a tiny non-significant residual inter ventricular communication. A repeat TTE and CCTA are planned in 6 months’ time as part of his outpatient follow-up.

## Discussion

Left ventricular free-wall or septal rupture, papillary muscle rupture, pseudo aneurysm and true aneurysm formation are widely considered the mechanical complications of acute myocardial infarction.^1,2^

A wide range of signs and symptoms, from dyspnea, hypotension and tachycardia to severe cardiogenic shock, may occur in case of post-infraction mechanical complications. Symptoms onset could be gradual in case of evolutive structural changes or immediate in case of hemodynamic compromise.^3^

In particular, post-infarction ventricular septal defect is defined as a defect in the ventricular septum that results from rupture of acutely infarcted myocardium.^4,5^ It has been shown that post-infarction VSD complicates approximately 1–2% of cases of acute MI.^6,7^ VSD occurs an average of 2–3 days after the infarction, but may occur any time in the first 2 weeks.^8^ Septal rupture, with interventricular left to right shunt, can lead to right ventricular failure, pulmonary edema until biventricular failure.^9^

Rarely, as in the case reported above, the infarcted muscular fibers can dissect forming a pseudoaneurym through the VSD within the subendocardial layer of the right ventricle.

In recent years, reperfusion therapies have led to a substantial reduction of the overall prevalence of post-infarction mechanical complications (less than 0.1% of patients). Despite optimal medical and surgical treatment, the mortality rates are still high, for patients presenting with such complication.^10,11^

Early diagnosis and aggressive approach to hemodynamic stabilization play a key role in the management of post-infarction VSD.^12^ Non-invasive imaging techniques, such as TTE or CCTA, are able to show morphological and functional cardiac modification consequent to VSD. In particular, TTE can reveal ventricular dysfunction and the left to right shunt by color Doppler function. On the other hand, CCTA offers a better anatomical characterization and can precisely determine dimension, position and extension of VSD and its effects on cardiac chambers; these proprieties make CCTA also useful for pre-operative planning. Thus, if combined, TTE and CCTA provide an early diagnosis of VSD and can improve patient’s management.^13^

Surgical repair or percutaneous device closure either as a bridge to definitive surgery, are considered the treatments of choice.

## Learning points

1. Acute myocardial infarction can lead to potentially fatal conditions: left ventricular free-wall or septal rupture, papillary muscle rupture, aneurysm or pseudo aneurysm are the most common AMI mechanical complications.
2. The extension of the VSD to the RV can lead to interventricular pathological communication or generate a cavity contained by a thin external layer of RV endocardium. Any defects of this layer can develop a pathological shunt.
3. Early diagnosis in fundamental for a better patient’s management: TTE and ECG-gated CCTA are considered reference standard.
4. Surgical repair is widely considered the treatments of choice, but perioperative mortality is still high.
